# Membrane Protein Activity Induces Specific Molecular Changes in Nanodiscs Monitored by FTIR Difference Spectroscopy

**DOI:** 10.3389/fmolb.2022.915328

**Published:** 2022-06-13

**Authors:** Federico Baserga, Antreas Vorkas, Fucsia Crea, Luiz Schubert, Jheng-Liang Chen, Aoife Redlich, Mariafrancesca La Greca, Julian Storm, Sabine Oldemeyer, Kirsten Hoffmann, Ramona Schlesinger, Joachim Heberle

**Affiliations:** ^1^ Department of Physics, Experimental Molecular Biophysics, Freie Universität Berlin, Berlin, Germany; ^2^ Department of Physics, Genetic Biophysics, Freie Universität Berlin, Berlin, Germany

**Keywords:** photoswitchable lipids, rhodopsin, cytochrome c oxidase, hydrogen bonding, lateral pressure, protein structural changes, lipid-protein interaction

## Abstract

It is well known that lipids neighboring integral membrane proteins directly influence their function. The opposite effect is true as well, as membrane proteins undergo structural changes after activation and thus perturb the lipidic environment. Here, we studied the interaction between these molecular machines and the lipid bilayer by observing changes in the lipid vibrational bands *via* FTIR spectroscopy. Membrane proteins with different functionalities have been reconstituted into lipid nanodiscs: Microbial rhodopsins that act as light-activated ion pumps (the proton pumps *Ns*XeR and *Um*Rh1, and the chloride pump *Nm*HR) or as sensors (*Np*SRII), as well as the electron-driven cytochrome *c* oxidase *Rs*C*c*O. The effects of the structural changes on the surrounding lipid phase are compared to mechanically induced lateral tension exerted by the light-activatable lipid analogue AzoPC. With the help of isotopologues, we show that the ν(C = O) ester band of the glycerol backbone reports on changes in the lipids’ collective state induced by mechanical changes in the transmembrane proteins. The perturbation of the nanodisc lipids seems to involve their phase and/or packing state. ^13^C-labeling of the scaffold protein shows that its structure also responds to the mechanical expansion of the lipid bilayer.

## Introduction

Membrane proteins reconstituted in lipids are often more stable and active than their detergent-solubilized counterparts (Shen et al., 2013) as lipid bilayers provide an environment that mimics the physiological conditions in the cell/organelle, especially when native lipids are used during the reconstitution (Robinson and Capaldi, 1977; Öjemyr et al., 2012b; Shen et al., 2013). Even though it would be optimal to always employ native lipids during preparation, it is often not possible due to technical reasons. Sometimes the native lipids might be simply not available, there might be no protocol to use them, or they might be unstable. For many proteins, it is also difficult to differentiate between the lipids necessary for activity and those needed for folding.

**FIGURE 1:**
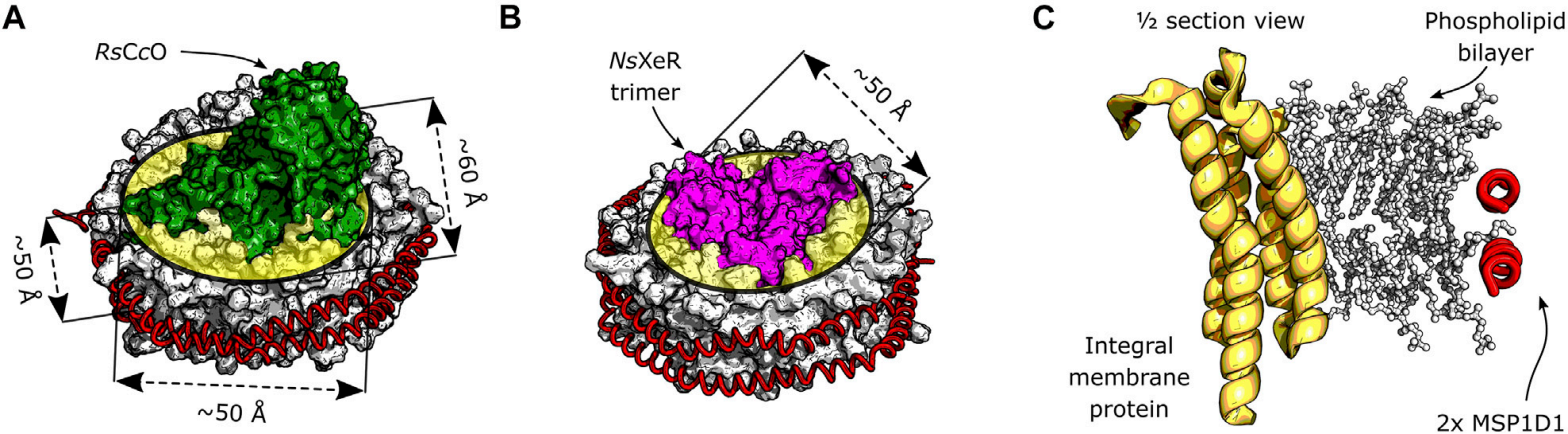
Schematic depiction of integral membrane proteins inserted in a nanodisc. The phospholipid bilayer is encircled by two α-helical membrane scaffold proteins MSP1D1. **(A)** Model of the *Rs*C*c*O assembly in lipid nanodiscs. Original crystal structures: PDB entries 1M56 (Svensson-Ek et al., 2002) and 6CLZ (Marcink et al., 2019). The approximate thickness of the bilayer, as well as the lateral dimensions of the inserted membrane protein are shown. **(B)** Model assembly of the *Ns*XeR trimer in lipid nanodiscs. Original crystal structures: PDB entries 6EYU (Shevchenko et al., 2017) and 6CLZ (Marcink et al., 2019). The trimer is approximately cylindrical, and its diameter is indicated. **(C)** Model cross section of a generic membrane protein reconstituted into a lipid nanodisc.

Common model membranes for reconstitution are lipid lamellar forms (Rand and Parsegian, 1989), for example monolayers, micelles, multilayer vesicles or the bilayer structure typical of cell membranes (Shen et al., 2013). Lateral pressure (Subirade et al., 1995) and curvature stress (Hong and Tamm, 2004; Bavi et al., 2014) in the membrane can have an effect on the local environment that is experienced by the proteins. Recently, the protocols for reconstituting membrane proteins in lipid nanodiscs were optimized and this setup became more popular (Bayburt et al., 2002; Bayburt and Sligar, 2010). The nanodisc reconstitution employs two membrane scaffold proteins (MSPs) that wrap around a lipid bilayer, as depicted in Figure 1. The lipid to transmembrane protein ratio is relatively low, usually in the order of 100 lipids per reconstituted protein. A single membrane protein or functional complex can be inserted per nanodisc, if an MSP of the appropriate length is selected. Using MSPs as a constraint for lipid bilayers allows them to act as a membrane mimetic (Siontorou et al., 2017), while avoiding long range force dissipation.

The resulting nanodisc assembly offers several benefits when compared to detergent-protein micelles or proteoliposomes. Due to their small size, nanodiscs exhibit less scattering than liposomes in the UV-visible range (Öjemyr et al., 2012b). Other advantages can resolve some technical issues as membrane proteins in nanodiscs are water soluble and higher concentrations than in detergent can be achieved, which may be advantageous for biophysical applications (e.g., recording EPR, NMR or IR spectra).

In this work, we reconstituted a variety of different integral membrane proteins into nanodiscs and studied the impact of their catalytic activity on the surrounding lipids. We employed microbial rhodopsins, which are transmembrane helical proteins acting as light sensors and enzymes, ion channels, and pumps (Ernst et al., 2014; Govorunova et al., 2017; Rozenberg et al., 2021). The results of these experiments reveal similarities to those of AzoPC which is an artificial lipid that exerts mechanical tension on the lipid bilayer upon photoisomerization (Morstein et al., 2021). Finally, the interaction of lipids with cytochrome *c* oxidase embedded in nanodiscs was studied to elucidate commonalities to this structurally and functionally very different membrane protein. Cytochrome *c* oxidase from *R. sphaeroides* (*Rs*C*c*O) is a model system for the aa_3_ oxidase family of heme-copper enzymes (García-Horsman et al., 1994; Pereira et al., 2001; Popovic et al., 2010). Some of the many observable changes in its cycle are a direct consequence of its redox reaction (Heibel et al., 1993; Kozuch et al., 2013); some are due to the interaction of the protein and cofactors (Sezer et al., 2017; Baserga et al., 2021); and some are related only to amino acid side-chains as derived by FTIR spectroscopy (Hellwig et al., 1999; Heitbrink et al., 2002; Iwaki et al., 2002). It is likely that many of these changes also entail structural rearrangements (Qin et al., 2009).

Using FTIR difference spectroscopy we show here how lipid-specific bands appear in different proteins and upon different trigger mechanisms. We hypothesize that these correlate with the packing state of the lipids in the nanodisc bilayer, reacting to mechanical changes in the reconstituted membrane proteins.

## Materials and Methods

### Protein Expression and Purification

Xenorhodopsin from *Nanosalina* sp. (*Ns*XeR) with a C-terminal 10xHis-tag was cloned into plasmid pET27b for protein expression in the *E. coli* strain C41(DE3). The protein was expressed and purified as described (Shevchenko et al., 2017). Cytochrome *c* oxidase (*Rs*C*c*O) carrying a C-terminal 6xHis-tag on subunit I was homologously expressed in the *Rhodobacter sphaeroides* strain JS100 (Shapleigh and Gennis, 1992) under aerobic conditions in Sistrom’s minimal medium (Cohen-Bazire et al., 1957), and purified by affinity chromatography in a comparable method to previous publications (Zhen et al., 1998; Sezer et al., 2015). The gene of halorhodopsin from *Nonlabens marinus* (*Nm*HR) (kindly provided by Dr. Przemyslaw Nogly, ETH Zurich) carrying a 10xHis-tag at the C-terminus, was cloned into pET27b. The plasmid was transformed into *E. coli* strain BL21-CodonPlus^™^ (DE3)-RP (Stratagene). The cells were grown at 37°C in BHI medium, supplemented with 50 μg/ml kanamycin. The protein was expressed and purified similarly to previous protocols (Tsukamoto et al., 2017). Sensory rhodopsin II from *Natronomonas pharaonis* (*Np*SRII) was expressed from a pET27b derivative, gifted by Prof. Martin Engelhard (MPI Dortmund), in *E. coli* strain BL21-CodonPlus^™^ (DE3)-RP. Expression was induced with 0.2 mM isopropyl β-D-1-thiogalactopyranoside (IPTG) at a cell density of OD_600_ = 0.8–1.0, in the presence of 12.5 μM all-*trans*-retinal. Protein purification was performed similarly to published protocols (Hohenfeld et al., 1999), but with different buffer compositions (breaking buffer: 0.5 M NaCl, 2 mM EDTA, 50 mM MES, pH 6; and elution buffer: 4 M NaCl, 50 mM MES, pH 4.5, 0.05% DDM). The eluant was subsequently equilibrated to pH 6. Rhodopsin 1 from the fungus *Ustilago maydis* (*Um*Rh1) was produced from a *Pichia pastoris* strain SMD1163 containing the gene in the vector pPIC9K under the control of an AOXI promoter. A hyperexpressing clone was generated by clone selection with increasing geneticin concentrations. The main culture was supplemented with all-*trans*-retinal addition and induced by methanol with subsequent purification as recently described (La Greca et al., 2022).

We generated and purified the 7xHis-tagged membrane scaffold protein MSP1D1 on the basis of published protocols (Bayburt et al., 2002; Denisov et al., 2004). MSP1D1 was expressed in *E. coli* grown in TB-medium supplemented with 50 μg/ml kanamycin from the plasmid pET28a-MSP1D1. 1 mM IPTG was used for induction. After centrifugation, bacterial pellets were resuspended in 300 mM NaCl, 40 mM Tris-HCl, pH 8.0, 1% Triton X-100. Cells were disrupted by several cycles of ultrasonification. The lysate was clarified by centrifugation and filtered (0.45 μm pore size). The filtered solution was purified by affinity chromatography *via* Ni-NTA column and size exclusion chromatography (Superdex 200 Increase 10/300 GL, Sigma-Aldrich) with a buffer exchange to 100 mM NaCl, 20 mM Tris-HCl, pH 7.4, 0.5 mM EDTA. Eluted fractions were analyzed by SDS-PAGE. The ^13^C-isotopically-labeled MSP1D1 (^13^C-MSP1D1) was obtained by growing in minimal medium M9 with ^13^C-glucose (Silantes GmbH).

### Nanodisc Assembly

Lipids, namely either 1,2-dipalmitoyl-sn-glycero-3-phosphocholine (DPPC, Avanti Polar Lipids, Inc.); uniformly ^13^C-labeled DPPC (Cambridge Isotope Laboratories), 1-stearoyl-2-[(E)-4-(4-((4-butylphenyl)diazenyl)phenyl)butanoyl]-sn-glycero-3-phosphocholine (AzoPC, Avanti Polar Lipids, Inc.), 1,2-dimyristoyl-sn-glycero-3-phosphocholine (DMPC, Avanti Polar Lipids, Inc.), a mixture of lipids from the membrane of *Escherichia coli* (*E. coli* polar extract with CA, PE and PG, Avanti Polar Lipids, Inc.), or 1-palmitoyl-2-oleoyl-glycero-3-phosphocholine (POPC, Avanti Polar Lipids, Inc.) were solubilized at 25 mM by sonication in 60 mM sodium cholate, 100 mM NaCl, 20 mM Tris-HCl at pH 7.5, until the solution became clear (30–60 min).

The detergent-solubilized (DDM) membrane proteins *Ns*XeR, *Rs*C*c*O, *Nm*HR, *Np*SRII and *Um*Rh1 were reconstituted into nanodiscs (see Figure 1 for a schematic view of the assembly) by incubation with the solubilized lipids and MSP1D1, with subsequent removal of detergent by incubation with SM2 Bio-Beads (Bio-Rad). The individual conditions and incubation procedures are summarized in Supplementary Table S1. After reconstitution, the nanodisc solution was purified from aggregates and from nanodiscs without membrane protein by centrifugation and size exclusion chromatography (Superdex 200 Increase 10/300 GL, Sigma-Aldrich). The eluted fractions were collected, concentrated, and their buffer was substituted with the final buffers specified below.

The following samples were used in the FTIR experiments: nanodiscs with 20% AzoPC and 80% DPPC (90 μM, in 140 mM NaCl, 3 mM KCl, 10 mM phosphate buffer, pH 7.2); *Ns*XeR-nanodiscs with either DPPC or DMPC lipids (100 μM, in 10 mM NaCl, 5 mM phosphate buffer, pH 7.4); *Nm*HR-nanodiscs in DPPC (100 μM, in 150 mM NaCl, 20 mM HEPES, pH 7.5); *Np*SRII-nanodiscs in DMPC (100 μM, in 30 mM NaCl, 5 mM MES, pH 6.0); *Um*Rh1-nanodiscs in POPC (200 μM, in 10 mM NaCl, 3.4 mM MOPS, pH 7.4); *Rs*C*c*O-nanodiscs with either *E. coli* polar lipids (PE + PG + CA) or DPPC lipids (50 μM, in 5 mM Tris-HCl, pH 8.0); nanodiscs with isotopically-labeled scaffold protein ^13^C-MSP1D1, containing 20% AzoPC and 80% DPPC (50 μM, in 140 mM NaCl, 3 mM KCl, 10 mM phosphate buffer, pH 7.2).

### FTIR Spectroscopy

The spectra of nanodiscs containing AzoPC and DPPC/^13^C-DPPC lipids were recorded on a Vertex 70v (Bruker) FTIR spectrometer in transmission configuration, at 4 cm^−1^ spectral resolution. Frequencies corresponding to wavenumbers higher than 4,000 cm^−1^ were optically filtered. About 30 μl of the nanodiscs in buffer were deposited on a BaF_2_ window, then dried under vacuum until opaque. Another BaF_2_ window enclosed the sample in a chamber encircled by a silicon-greased spacer of 0.5 mm thickness. For the difference spectra, single channel spectra (500 scans each) of the sample were recorded in darkness after 5 min illumination of either blue (450 nm LED, 90 mW/cm^2^) or UV (365 nm LED, 3 mW/cm^2^) light. Seven illumination/measurement cycles were repeated for each LED light, alternating the illumination between blue and UV. The difference spectra of the light switching were obtained for each spectrum (starting from the second) by using the previous single channel as a reference spectrum. Finally, all difference spectra of the switching in each of the two directions were averaged.

Light-induced difference spectra of *Ns*XeR, *Np*SRII, and *Um*Rh1 in nanodiscs and detergent were recorded in transmission configuration using a Vertex 80v FTIR spectrometer (Bruker) as previously described (La Greca et al., 2022). Spectra of *Nm*HR in nanodiscs and detergent were recorded in ATR configuration. Approximately 4 μl of *Nm*HR in solution were deposited onto a ZnS ATR cell and dried for 15 min. The sample was illuminated using a 525 nm LED, and light minus dark difference spectra were obtained by using the dark single channel as a reference spectrum. Spectra of *Rs*C*c*O in nanodiscs and detergent were recorded on a Tensor 27 (Bruker) FTIR spectrometer using a Si ATR cell. Approximately 2 μl of oxidized *Rs*C*c*O were deposited on the ATR crystal, dried with N_2_, reduced with ∼10 equivalents of Na_2_S_2_O_4_ and dried again. The protein film was rehydrated in H_2_O aerosol and exposed to a flow of CO, yielding the fully-reduced, CO-bound state. Our setup (Senger et al., 2018) allowed the rapid exchange of the atmosphere over the protein film with 40% O_2_, triggering the oxidation of *Rs*C*c*O. N_2_ was used as a carrier over the whole experiment. Every difference spectrum is the result of a different number of co-additions of pre-trigger and post-trigger absolute spectra that maximized SNR (Stripp, 2021), ranging from a minimum of 20 scans to a maximum of 500.

All experiments were performed under ambient conditions (room temperature and pressure).

## Results

### Changing Lateral Tension in Nanodiscs by Light Switching of AzoPC

**FIGURE 2:**
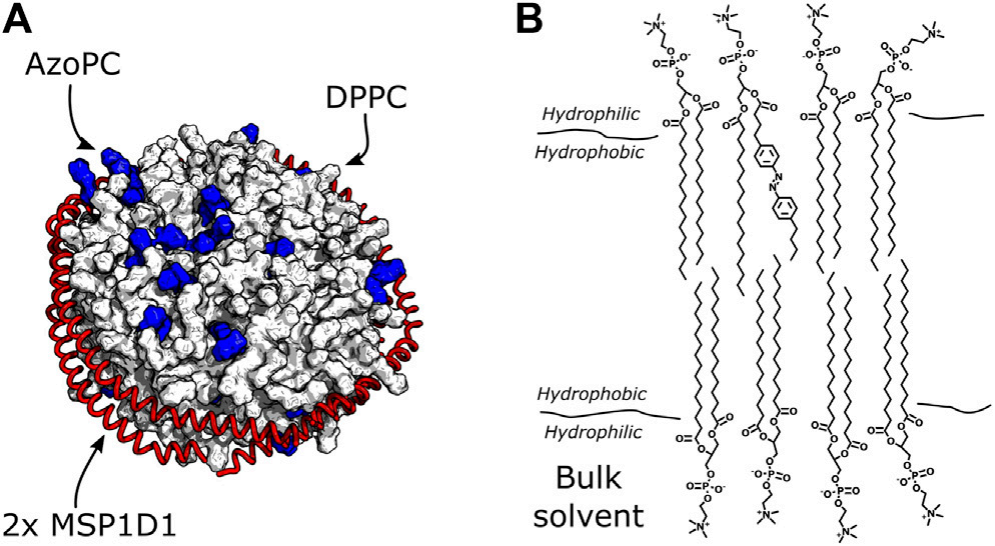
Nanodiscs with DPPC and AzoPC. **(A)** Schematic depiction of nanodiscs with 80% DPPC and 20% AzoPC. The AzoPC molecules are highlighted in blue, and their random distribution in the bilayer is simply illustrative of one configuration they could exhibit. **(B)** Chemical structure of one AzoPC molecule inserted into the bilayer of a nanodisc containing DPPC. The aliphatic tails of the lipids reside in the hydrophobic membrane environment, while their glycerol groups are in contact with the bulk solvent.

**FIGURE 3:**
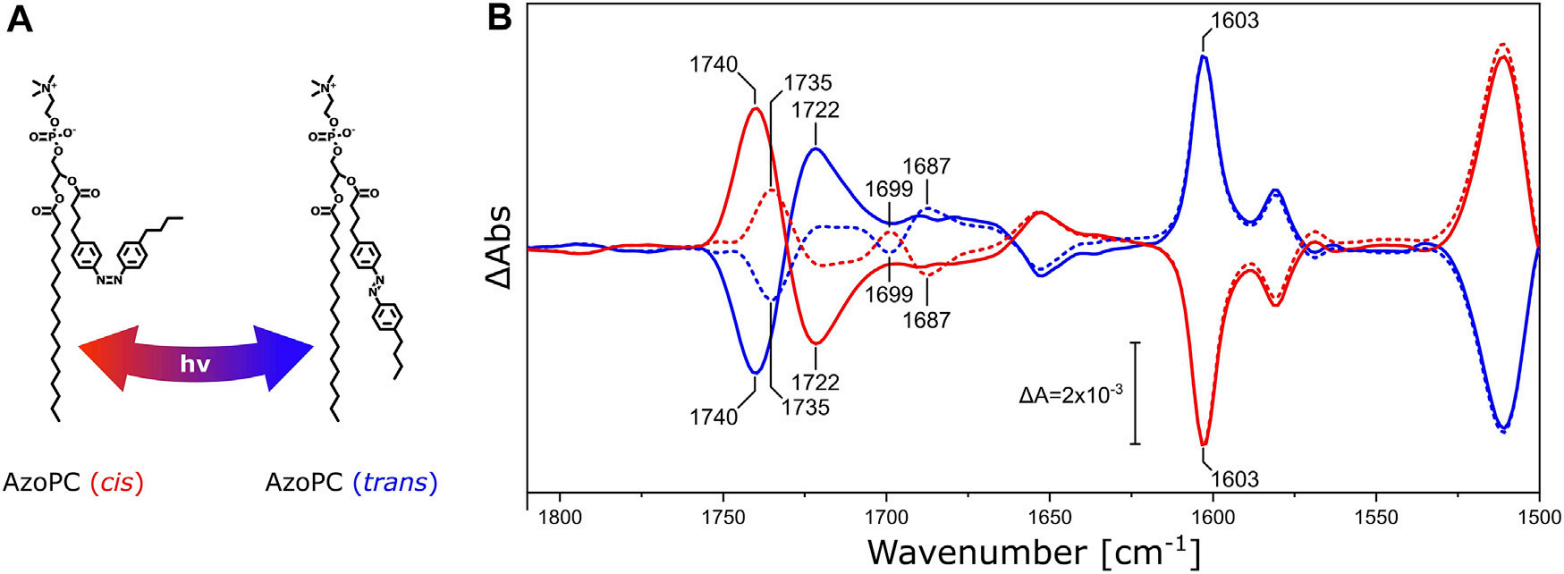
DPPC ester response to AzoPC isomerization switch. **(A)** Chemical structures of *cis* and *trans* AzoPC upon isomerization with the use of 365 and 450 nm light. **(B)** FTIR difference spectra of nanodiscs composed of 80% DPPC and 20% AzoPC upon transition of AzoPC from *trans* to *cis* (red) and from *cis* to *trans* (blue). The spectra of nanodiscs containing native DPPC are shown as solid lines, while the spectra of nanodiscs with ^13^C-DPPC are shown as dashed lines. Difference spectra have been scaled to the ring mode of AzoPC peaking at 1,603 cm^−1^ (Wu et al., 1999). Other significant band positions are labeled with their respective frequencies.

Nanodiscs are small bilayer patches of about 150/200 lipids, surrounded by two scaffold proteins (Figure 2A). To understand if mechanical force exerted from embedded molecules in the bilayer can be sensed by the surrounding lipids, we replaced 20% (molar) of the nanodisc lipids with AzoPC (Figure 2B). This photoactive lipid molecule contains an azobenzene-substituted sn-2 alkyl chain which undergoes isomerization upon illumination (Morstein et al., 2021) (see Figure 3A). *Trans* to *cis* isomerization is induced by UV light with a wavelength of 365 nm and exerts lateral pressure on the membrane (Pritzl et al., 2020), serving as mechanical perturbation of the bilayer. The photoreaction is reverted by illumination of the *cis* state of AzoPC by 450 nm light to form the initial *trans* isomer.

Pure AzoPC exhibits a strong absorption band from the lipid ester ν(C=O) vibrational band at 1,735 cm^−1^ (Supplementary Figure S1A). The light-induced difference spectrum of nanodiscs between the two isomeric states (Figure 3B) exhibits a pair of bands at 1,740 and 1,722 cm^−1^, respectively, with a positive and a negative band in the *trans* to *cis* transition, and with the opposite signs *vice versa*. The esters of DPPC are also absorbing in this frequency window and the lipid mixture in the nanodisc shows the sum of both absorptions. In order to disentangle the ν(C=O) vibrational bands of DPPC and AzoPC in the lipid mixture of the nanodisc, we used the ^13^C-isotopologue of DPPC (Blume et al., 1988). It is known that the ν(C=O) ester band of phosphatidylcholines (PCs) is red-shifted from 1737 to 1693 cm^−1^ by ^13^C-isotopic substitution (Blume et al., 1988; Lewis et al., 1994), (Supplementary Figure S1B). It is evident from the FTIR difference spectra (Figure 3B) that the band at 1,740 cm^−1^ shifts into two separate bands at 1,735 cm^−1^ and 1,699 cm^−1^, with the former assigned to the ν(^12^C=O) vibrational mode of AzoPC and the latter to the ν(^13^C=O) of DPPC. As an internal control, the reverse reaction from *cis* to *trans* AzoPC exhibits mirror-like spectra. The changes in absorption are much larger for AzoPC than in DPPC despite the 4-fold excess of DPPC. That AzoPC exhibits intense changes is expected, as the isomerization occurs directly next to its ester moiety, while the esters of DPPC only indirectly report on the lateral pressure changes. Still, adding the intensities of the bands at 1,735 cm^−1^ and 1,699 cm^−1^ (dashed lines in Figure 3B) does not equal the intensity of the band at 1,740 cm^−1^ (continuous lines in Figure 3B). We assume that these absorption changes might originate from a slightly different lipid ratio between the two samples, which is difficult to control experimentally even when using identical preparation protocols.

It is evident that the geometric changes in the glycerol backbone of DPPC are caused by mechanical stress exerted on the lipid bilayer *via* isomerization of AzoPC. Clearly, the scaffold proteins acts as a constraint to the bilayer system, allowing the build up of pressure. This approach facilitates activating mechanosensing proteins (Cox et al., 2019) by light. A more fundamental question is whether integral membrane proteins also induce mechanical stress in the nanodisc bilayer when they undergo structural changes associated with catalytic activity.

### Lipid-Protein Interactions of Nanodisc-Reconstituted *Ns*XeR

We started our studies on lipid-protein interactions utilizing microbial rhodopsins. (Ernst et al., 2014), a large protein family that can act as light sensors and enzymes, ion channels, or pumps (Govorunova et al., 2017; Rozenberg et al., 2021). With the possibility to probe protonation states of amino acids, FTIR spectroscopy has contributed to a large extent in understanding the molecular mechanism of these molecular machines (Radu et al., 2009).

**FIGURE 4:**
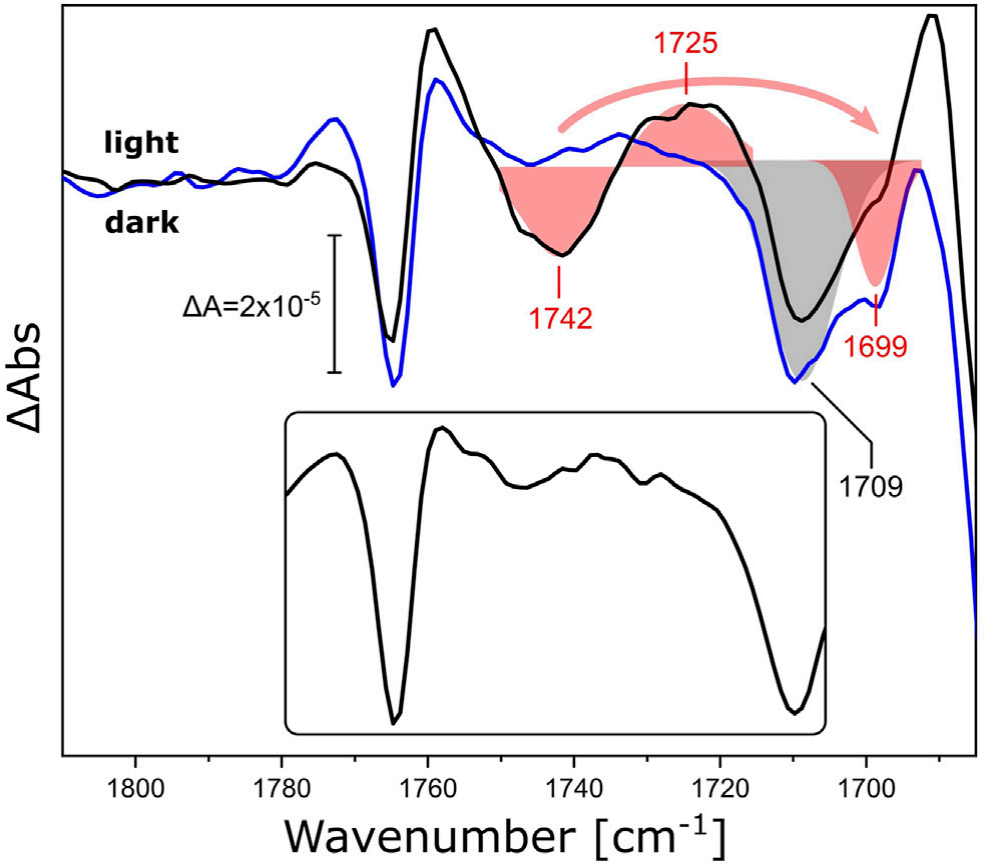
Light-induced FTIR difference spectra of *Ns*XeR in the carbonyl stretching region. The light-minus-dark difference spectra refer to *Ns*XeR reconstituted in nanodiscs with DPPC (black) and uniformly ^13^C-labeled DPPC (blue). The ν(C=O) ester bands of the lipids were fitted by Gaussians and are shown as red shaded areas with centers at 1,742/1,725 cm^−1^ for ^12^C-DPPC and 1,699 cm^−1^ for the ^13^C isotopologue. The grey shaded area corresponds to a band with a minimum at 1,709 cm^−1^ that is invariant to ^13^C-labeling. The inset shows the light-minus-dark spectrum of DDM-solubilized *Ns*XeR under the same experimental conditions.

As a representative of this family, we reconstituted xenorhodopsin from *Nanosalina* sp. (*Ns*XeR) in lipidic nanodiscs (Supplementary Figure S2). Continuous illumination of *Ns*XeR generates a photostationary state with major contribution from the longest-lived intermediate state (Supplementary Figure S3) (Weissbecker et al., 2021). The corresponding light minus dark FTIR difference spectrum in the ν(C=O) region (Figure 4, black line) shows a sigmoidal band with a negative peak at 1,742 cm^−1^ and a positive peak at 1,725 cm^−1^, that are absent in the difference spectrum of the detergent-solubilized sample (Figure 4, inset). Substituting DPPC lipids in the nanodiscs by their ^13^C-labeled isotopologue (Figure 4, blue line) selectively affects the aforementioned feature, leaving all other bands unchanged. While the negative peak shows a characteristic frequency downshift from 1,742 to 1,699 cm^−1^, it is not possible to identify the corresponding positive peak at lower frequencies due to the overlap with stronger protein bands. The shoulder at 1,699 cm^−1^ overlaps with the negative band peaking at 1,709 cm^−1^. The latter band may be due to the ν(C=O) band of a protonated carboxylic acid side chain (Lorenz-Fonfria, 2020) and will be assigned in future experiments on variants where candidate aspartic and glutamic acids are exchanged.

While isotopic substitution and solubilization in detergent allow for an unequivocal assignment of the difference bands at 1,742 cm^−1^ and 1,725 cm^−1^ to the ester ν(C=O) vibration of DPPC, it is interesting to note that the same frequencies have been observed for the ester carbonyl of DPPC upon light switching of AzoPC (Figure 3, continuous lines). Thus, we conclude that the structural changes of *Ns*XeR (associated with proton pumping) lead to structural changes in the hydrogen-bonded environment of the lipid esters that are almost identical to those originating from the mechanical switching using AzoPC as a tool.

### Lipid-Protein Interactions of Other Microbial Rhodopsins

**FIGURE 5:**
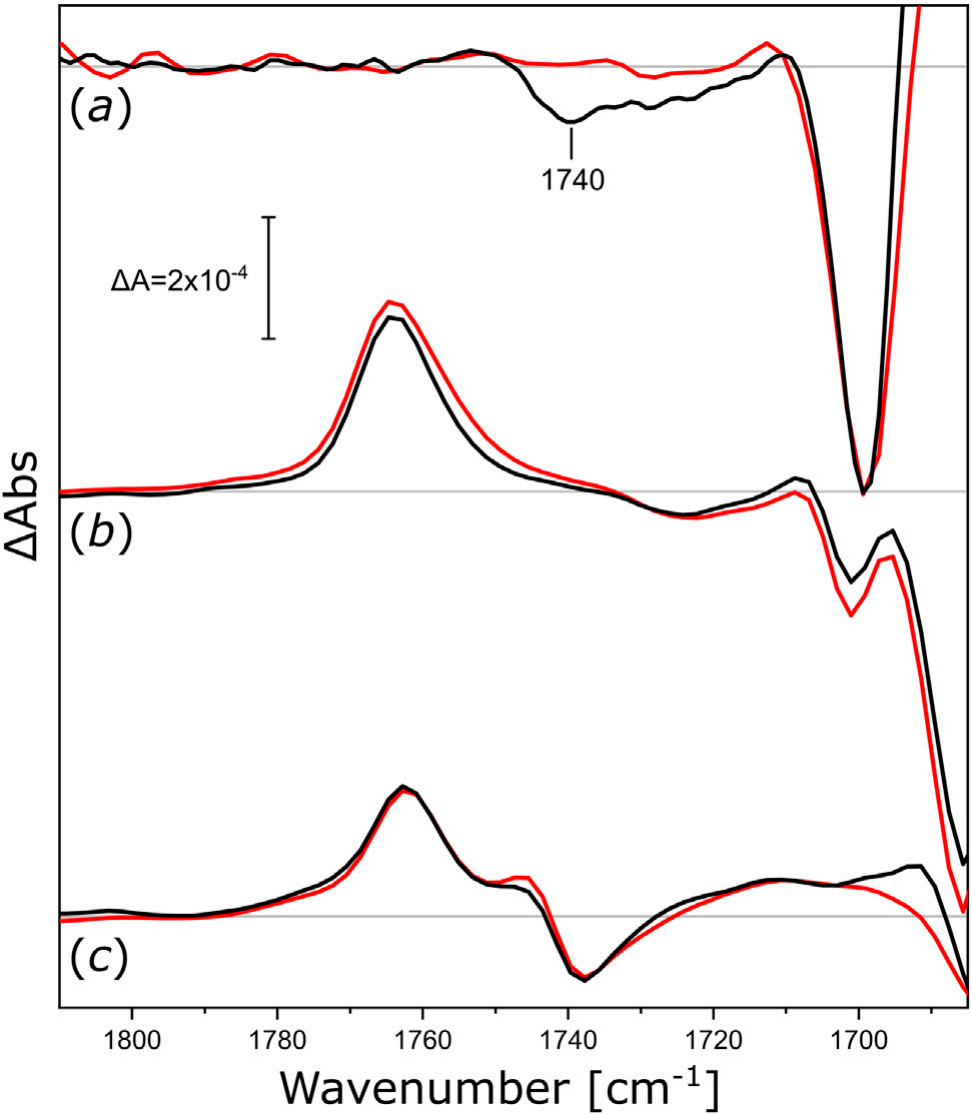
Light-induced FTIR difference spectra of integral membrane rhodopsins reconstituted in different nanodiscs. (*a*) Difference spectra of *Nm*HR reconstituted in DPPC nanodiscs (black) and *Nm*HR solubilized with DDM (red) upon illumination with 525 nm LED light. (*b*) Difference spectra of *Np*SRII reconstituted in DMPC nanodiscs (black) and solubilized with DDM (red) upon illumination with 505 nm LED light. (*c*) Difference spectra of *Um*Rh1 reconstituted in POPC nanodiscs (black) and solubilized with DDM [red, data taken from (La Greca et al., 2022)] upon illumination with 525 nm LED light. All spectra in this figure are normalized to the ethylenic modes of the retinal in the spectrum of solubilized *Np*SRII. Zero lines are shown in grey, discussed bands are labeled.

After the proton-pumping *Ns*XeR, we studied other microbial rhodopsins exhibiting different functionalities. We reconstituted *Nm*HR (the chloride pump from *N. marinus*), *Np*SRII (the sensory rhodopsin II from *N. pharaonis*), and *Um*Rh1 (the proton pump 1 from *U. maydis*) in nanodiscs to check if their activity induced similar changes in the surrounding lipids. The light-induced FTIR difference spectra (Figure 5) of *Nm*HR reconstituted in DPPC nanodiscs display a broad negative band at around 1,740 cm^−1^ (Figure 5, black trace in *a*) which is absent upon solubilization in detergent (red trace). Therefore, we can deduce that the light-induced activity of *Nm*HR also leads to changes in the glycerol backbone of the surrounding lipids. The light-induced change in absorption of the ester ν(C=O) vibration seems to be smaller for *Nm*HR as for *Ns*XeR (Figure 4), but quantification is less reliable with FTIR difference spectroscopy.

The assemblies of *Np*SRII in DMPC nanodiscs (Figure 5, spectra in *b*) and *Um*Rh1 in POPC nanodiscs (Figure 5, spectra in *c*) exhibit vibrational changes that are very similar to the detergent-solubilized samples. The absence of observable ester-specific bands at around 1,740 or 1,720 cm^−1^ leads us to conclude that either the functional activities of these two microbial rhodopsins or their photo-accumulated intermediate states do not exert any observable mechanical stress to the surrounding lipid bilayer. In any case, these experiments demonstrate that, under the conditions of our nanodisc reconstitutions, some of these proteins are not able to undergo structural changes provoking responses in the ester band of the nanodisc lipids during their photoexcitation. Further studies may be required to clarify whether different lipids would provide a better match for *Np*SRII and *Um*Rh1, possibly resulting in signal transduction from the activated protein to the surrounding lipids.

### Catalytic Structural Changes of Cytochrome *c* Oxidase Also Affect the Surrounding Lipids

So far, we have considered systems that exert stress on the surrounding lipid phase after photonic excitation. Using cytochrome *c* oxidase (C*c*O), the terminal complex of the respiratory chain (Brzezinski and Gennis, 2008), we will show that the impact on the lipid bilayer applies to an electron-driven membrane protein as well, in this case even exhibiting a complex quaternary structure comprising four subunits (Svensson-Ek et al., 2002). Bacterial C*c*Os have been reconstituted in lipidic nanodiscs (Öjemyr et al., 2012b; Kolbe et al., 2021) and present an excellent system to study such interactions. Some of the redox-induced changes in C*c*O are already known to be modulated by the membrane (Öjemyr et al., 2012a), making reconstitution protocols all the more important.

**FIGURE 6:**
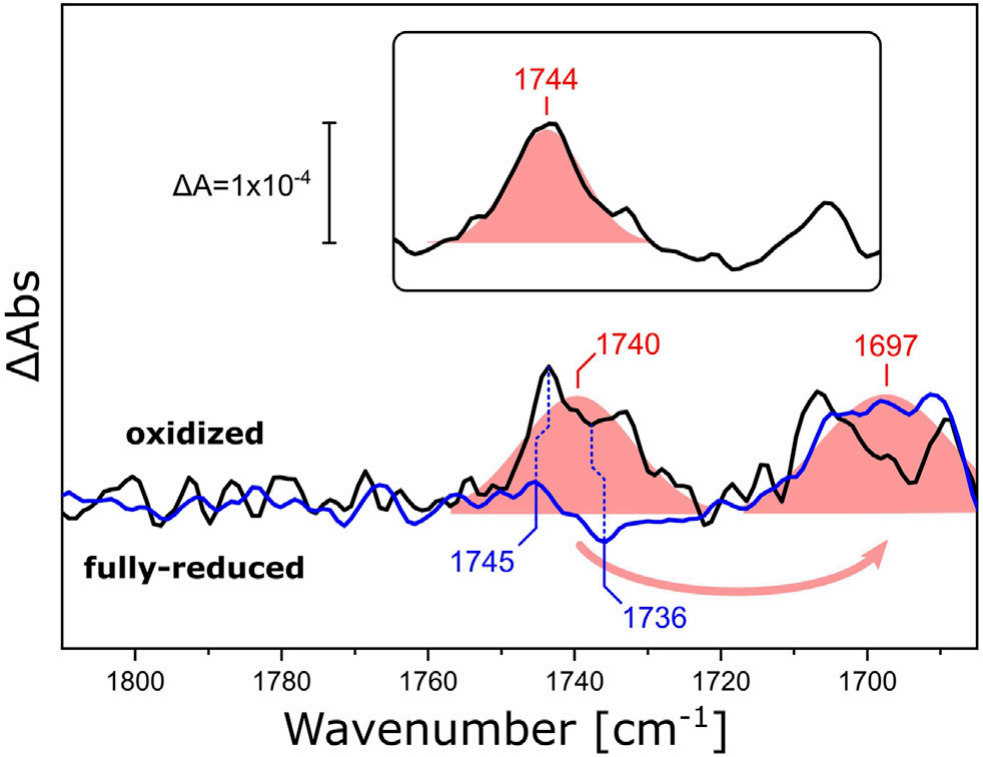
FTIR difference spectra of *Rs*C*c*O reconstituted in nanodiscs with different lipids. Difference spectra of O_2_-oxidized *Rs*C*c*O minus chemically reduced, CO-bound, *Rs*C*c*O, reconstituted in nanodiscs containing either DPPC (black line) or ^13^C-DPPC (blue line). The inset shows the difference spectra of *Rs*C*c*O reconstituted in nanodiscs containing *E. coli* polar lipids. Gaussian fits are shown as shaded red areas. Band positions from the peak fits are labeled. Blue vertical dashed lines highlight the high- and low-frequency components of the C=O vibrational band of E286. The spectra were scaled to yield similar absorbances for the fitted bands.

We investigated how different lipid reconstitutions of *Rs*C*c*O reacted to exposure to O_2_. We reconstituted *Rs*C*c*O in nanodiscs using *E. coli* polar lipids, DPPC, and ^13^C-labeled DPPC. After sample reduction and rehydration (Supplementary Figure S4), the reaction with O_2_ was induced by quickly switching the aerosol gas composition from CO to O_2_ in N_2_ carrier (the O_2_ pulse lasted for approximately 5 s). FTIR difference spectra (Supplementary Figure S5) that reflect the structural and electronic changes upon the catalytic reaction of C*c*O were recorded *in situ*. Figure 6 shows the region of the ester ν(C=O) vibration. Evidently, a positive band at 1,740 cm^−1^ appears upon the oxidation of fully reduced *Rs*C*c*O. Isotopic substitution of DPPC for ^13^C-DPPC lipids results in a characteristic band shift of 43 cm^−1^ (Hübner and Mantsch, 1991). These experiments provide evidence that the oxygen reaction of *Rs*C*c*O in nanodiscs influences the ester C=O of the lipids in the bilayer (Blume et al., 1988; Wong and Mantsch, 1988). The sigmoidal-shaped band feature at 1,745/1,736 cm^−1^ that overlays the difference spectra of *Rs*C*c*O in DPPC corresponds to the ν(C=O) vibration of the residue E286 undergoing a change in hydrogen bonding (Nyquist et al., 2001; Nyquist et al., 2003). This band feature is devoid of overlap in the difference spectrum of C*c*O reconstituted in ^13^C-DPPC, and matches the difference spectrum of the detergent-solubilized enzyme (Supplementary Figure S6). It is not possible to clearly observe the bands corresponding to E286 when *Rs*C*c*O is reconstituted in *E. coli* polar lipids (inset in Figure 6), possibly because of the relative intensity of the lipid response, or the slightly shifted frequency of the ester ν(C=O) to 1,744 cm^−1^.

We have previously reported a similar behavior of lipid ester bands in spectroelectrochemical titrations of *Rs*C*c*O embedded in hydrated DPPC layers (Baserga et al., 2021). However, it was impossible to determine whether this contribution originated from protein activation or from the external electric field applied to the protein-lipid film (Zawisza et al., 2007). The band positions at ∼1,740 and ∼1,699 cm^−1^ for *Rs*C*c*O in DPPC and ^13^C-DPPC, respectively (Supplementary Figure S7) match the data presented in Figure 6.

In essence, our FTIR difference experiments on *Rs*CcO show a band at 1,740 cm^−1^ that is assigned to the ester ν(C=O) vibration of the surrounding DPPC. We can theorize that this electron-driven proton pump also undergoes structural changes that are transduced to the adjacent lipid bilayer, much like the light-activated rhodopsins discussed above, but provoking a different response.

### Response of the Lipid Alkyl Chains to Mechanical Stress

**FIGURE 7:**
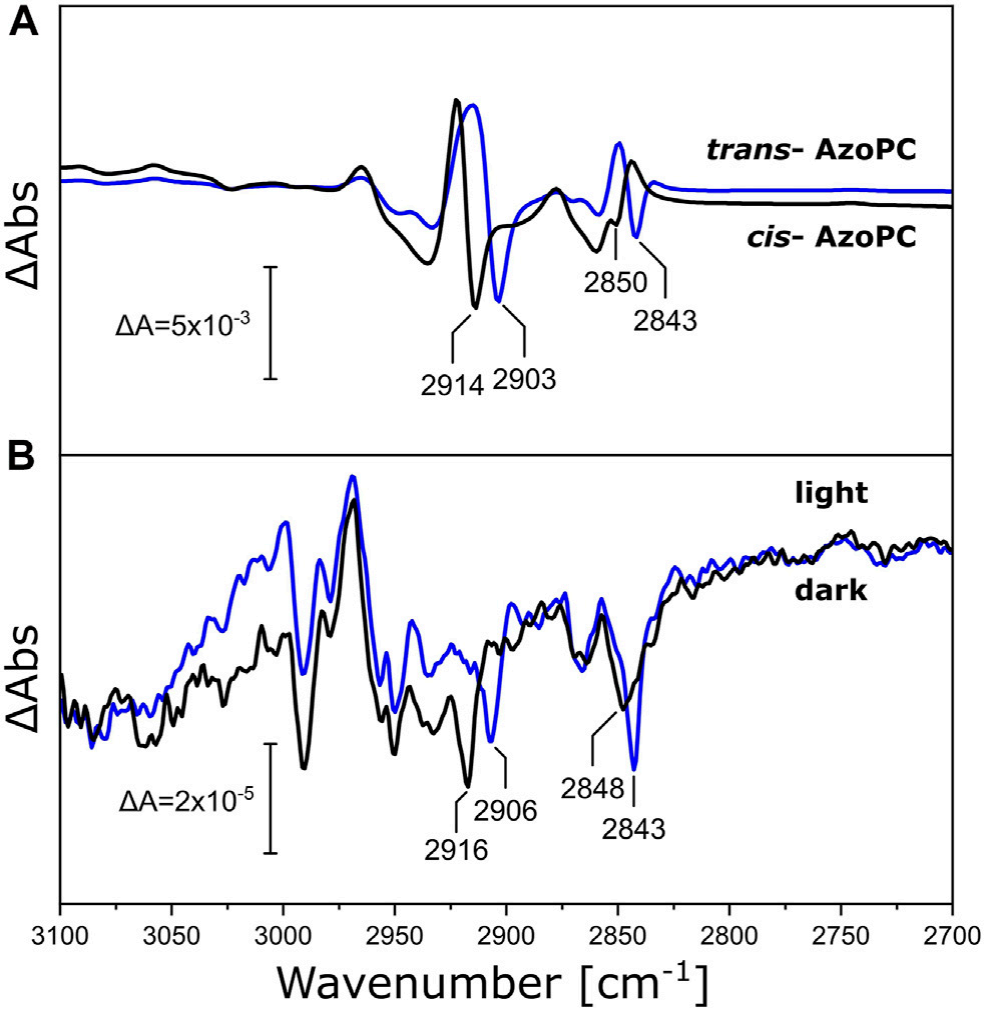
Changes in the alkyl chains of DPPC upon applied stimuli. **(A)** C-H stretching region of the FTIR difference spectra of nanodiscs supplied with 20% AzoPC and 80% DPPC (black line) or 80% ^13^C-DPPC (blue line) upon 450 nm LED illumination. Difference spectra are normalized to the peak absorbance at 2,914/2,903 cm^−1^. **(B)** FTIR difference spectra of *Ns*XeR in nanodiscs with either DPPC (black line) or ^13^C-DPPC (blue line) upon 525 nm LED illumination. These spectra are not scaled nor baseline corrected. Peak positions identifiable in both systems within a margin of ±2 cm^−1^ are labeled.

Above, we have measured the protein-lipid interaction by recording the ester ν(C=O) vibration of DPPC in the nanodisc bilayer in the range of 1,685–1,810 cm^−1^, but the fatty acid long chains are also affected by the mechanical stress induced by the conformational changes of *Ns*XeR or AzoPC. Inspecting the frequency range at higher energies (3,100—2,700 cm^−1^, Figure 7), we observe bands that are shifted by the increased mass of the ^13^C-DPPC isotopologue upon the photoreaction of both the AzoPC-supplied nanodiscs and the *Ns*XeR nanodiscs. In particular, two bands exhibit a 5—10 cm^−1^ redshift in their methylene ν(CH_2_) stretching frequencies upon ^13^C-labelling of DPPC. Namely, peaks at 2,843, ∼2,849, ∼2,905 and ∼2,915 cm^−1^ consistently show the same changes in both photoreactions. Among all the peaks that can be found in Figure 7, only these correspond well to the band positions observed in the absolute spectra of the samples (Supplementary Figures S1, S2).

### Response of the Nanodiscs’ Scaffold Protein to Light-Induced Mechanical Stress

**FIGURE 8:**
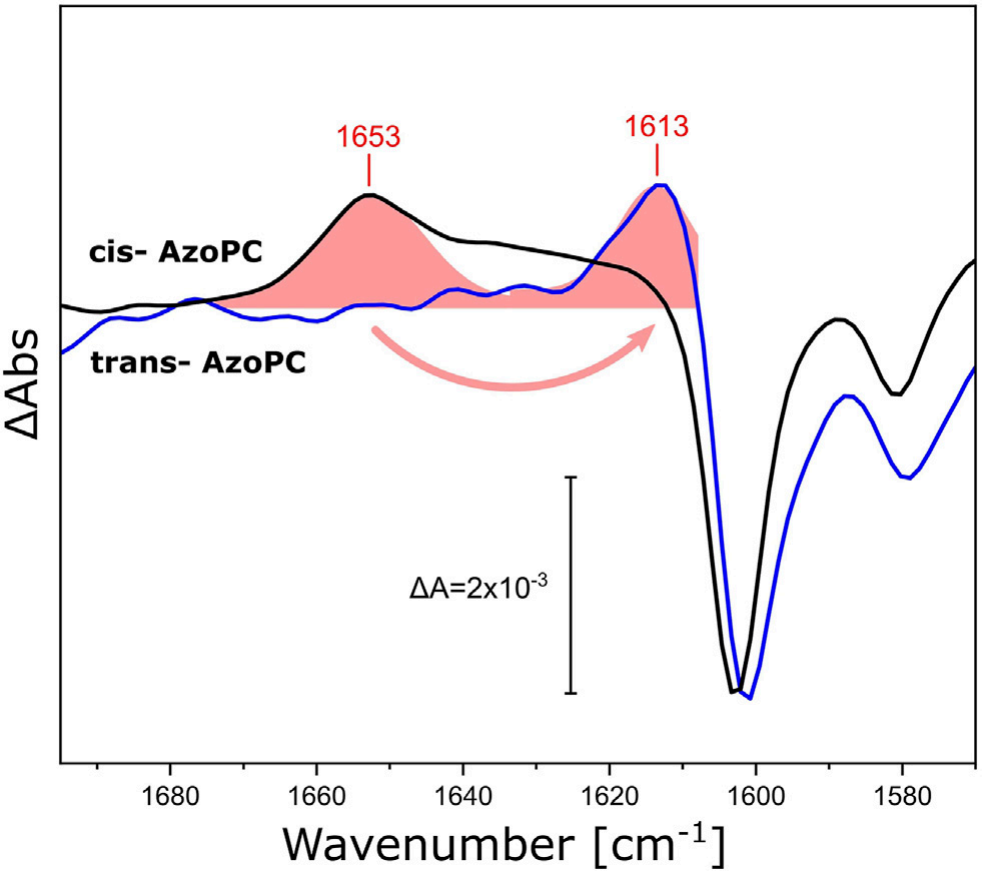
Response of the amide I vibrational mode of MSP1D1 upon AzoPC isomerization. FTIR difference spectra recorded on MSP1D1 nanodiscs containing 80% DPPC and 20% AzoPC, upon the transition of AzoPC from *trans* to *cis* using 365 nm light. The spectra relative to the non-labeled scaffold protein are identical to those presented in Figure 3B (black line). The spectra of the nanodisc assembly with uniformly ^13^C-labelled MSP1D1 (blue line) are normalized to the ∼1,603 cm^−1^ peak. The amide I difference bands of the scaffold proteins were fitted by Gaussians (shaded red areas), and the positions of the peak maxima are labeled.

We demonstrated that the DPPC lipids in the nanodiscs bilayer respond by changes in their ester as well as their alkyl chains when stress is applied to the assembly, either in the form of lateral pressure by AzoPC isomerization or responding to the activation of reconstituted proteins. The scaffold protein of the nanodiscs was utilized as a physical barrier for the volume expansion to generate lateral pressure. Yet, the scaffold protein is a helical polypeptide which is not rigid, but more akin to a flexible wall and expected to respond to lateral tension. Thus, we re-analyzed our light-induced FTIR difference spectra recorded on nanodiscs supplemented with AzoPC (Figure 3) and compared these to the ones recorded on uniformly ^13^C-labeled scaffold protein (^13^C-MSP1D1, Figure 8). Evidently, isotope labeling downshifts the broad band from 1,653 cm^−1^ by 40 cm^−1^, agreeing well with the expected behavior of the amide I mode (Haris et al., 1992). Given the overlap of the amide I band in ^13^C-MSP1D1 and the AzoPC ring breathing mode at 1,603 cm^−1^, the latter appears slightly redshifted (blue spectrum in Figure 8).

The scaffold protein MSP1D1 is amphipathic and α-helical (Bayburt et al., 2002), which facilitates the interpretation of its amide I response to the stress induced by AzoPC isomerization. Switching AzoPC from *trans* to *cis* increases the lateral pressure of the bilayer (Pritzl et al., 2020): we suggest that this force is transduced to the scaffold protein, resulting in the positive amide I band. The detailed mechanism by which only a positive amide I band is recorded, in contrast to a sigmoidal-shaped response that would indicate structural changes, remains unresolved.

## Discussion

We reconstituted transmembrane proteins into lipidic nanodiscs. With rhodopsins of different functionalities (ion pumps and sensors), and cytochrome *c* oxidase as a representative of a multi-subunit electron-coupled proton pumps, we studied the mechanical stress induced on the nanodisc by the stimulated structural change of these proteins. The responses have been compared to those induced by the light-induced isomerization of AzoPC, a derivative of PC with an azobenzene moiety in one of the aliphatic chains of the lipid. Using uniformly ^13^C-labeled isotopologues of the lipid DPPC and of the scaffold protein MSP1D1, we monitored the molecular changes in the lipid phase with FTIR difference spectroscopy by triggering structural changes in the proteins and in the azolipids. With this work, we also provided evidence that the induced mechanical stress on the lipid bilayer is propagated to the scaffold protein encircling the lipid bilayer.

Nanodiscs were employed as biomimetic membranes and integral membrane proteins were reconstituted into the lipid bilayer. We showed that the lipids in the nanodiscs exhibit changes in the ν(C=O) vibrational band of their ester groups and in the ν(CH_2_) band of their alkyl chains. FTIR spectroscopy is an exquisite tool to derive the protonation states of aspartic and glutamic side chains by analyzing their ν(C=O) stretching vibrations. However, the analysis of these bands in membrane proteins reconstituted in nanodiscs is hampered, due to the fact that the frequencies of carboxylic acid side chains overlap with the lipid ester vibration. Using ^13^C-isotopologues of the lipids downshifts the latter by more than 40 cm^−1^, alleviating the spectral overlap.

Hydration plays a critical role in the lipid ester bands, and at least two H_2_O molecules per lipid form hydrogen bonds (Fowler Bush et al., 1980). The band observed for the decoupled ν(C=O) in the difference spectrum of Figure 3B indicates that the DPPC lipid esters experience a stronger H-bonding to the bulk solvent (Blume et al., 1988) when AzoPC is in its *trans* state, rather than in the *cis* state. This correlates with lower lateral pressure reducing the hydrogen bonding of the lipid headgroups. Changes in H-bonding are related to a different orientation of the phospholipid head groups (Hübner and Mantsch, 1991; Pfeiffer et al., 2003) and indicative of phase changes (Lewis and McElhaney, 2013). Since the hydration of the bilayer changes with its phase state, temperature and hydration are strongly linked (Carlson and Sethna, 1987; Pfeiffer et al., 2003). Likewise, the thickness of the bilayer will change in response to changes in hydration, as the alkyl chains become more ordered when the bilayer is dehydrated (Dvinskikh et al., 2005).

The phase transition L_β_/L_α_ of DPPC, entailing the melting of the alkyl chains of the PCs, takes place at ∼41°C in liposomes (Biltonen and Lichtenberg, 1993). DPPC lipids integrated into nanodiscs exhibit a slightly higher transition temperature of ∼44°C, and are characterized by a thin boundary layer at the MSP interface in which the lipids have a higher predisposition to be found in their gauche conformer (Denisov et al., 2005). In binary lipid mixtures, the presence of 20% AzoPC may influence the thermodynamics of the DPPC bilayer. All our experiments were performed at ∼23°C which is significantly lower than the phase transition temperature of DPPC (Denisov et al., 2005). It is reasonable to assume that, even after “doping” with AzoPC, the DPPC lipids inside the nanodiscs are in the gel phase L_β_, and that the addition of AzoPC does not induce a phase transition upon their isomerization, as was shown by NMR spectroscopy (Doroudgar et al., 2021). The lipid mixture of 20% AzoPC and 80% DPPC in our nanodisc preparation may lead to phase separation depending on the isomerization state of AzoPC, as has been observed for binary mixtures of AzoPC with DPhPC [1,2-diphytanoyl-sn-glycero-3-phosphocholine (Urban et al., 2018)]. The sigmoidal band shapes of the ν(CH_2_) stretching modes (Figure 7A), showing an upward shift in both symmetric and asymmetric modes (see Supplementary Figure S1) upon illumination, point to the isomerization of AzoPC from *cis* to *trans* inducing the phase transition to L_α_ for DPPC (Lewis and McElhaney, 2013). The inverse reaction is prompted by *trans* to *cis* isomerization, proving that this phase transition is induced by changes in lateral pressure rather than local heating originating from the azobenzene absorbers. The response of the fatty acid ester bands in the same experiment (Figure 3B) is coherent with this proposal.

The insertion of a membrane protein into nanodiscs with PC lipids does not necessarily lend itself to the same interpretation. The effective number of lipids per nanodisc is much smaller in this case, and lipid-lipid interactions are enhanced by steric hindrance as an effect of the protein-occupied volume. Small effects that might involve boundary lipids are magnified by the high contact area to lipid ratio. It is also known that the insertion of a peptide or a membrane protein into a PC bilayer lowers the melting temperature of the surrounding lipids, and that tighter lipid packing often form crystalline or quasi-crystalline lipid phases (Lewis and McElhaney, 2013). The ester bands of PCs are elegant reporters for identifying the differences between low and high temperature gel states and liquid-crystalline state (Mantsch et al., 1985). This is because the H-bonding environment that they sense is directly related to the water and headgroup structural distribution (Binder, 2007), with the geometric orientation of the headgroups in relation to the alkyl tails also being crucial (Zawisza et al., 2007). This information actually leads to a similar interpretation of the *Ns*XeR data as for the AzoPC/DPPC system. The ester band (Figure 4) is also redshifted, and the peak positions match those of Figure 3. The alkyl chains of DPPC in both nanodiscs with *Ns*XeR or AzoPC (Figure 7) also react similarly, and coherently with their ester bands. Thus, we interpret this results in the same way: Illumination of *Ns*XeR induces a phase transition in DPPC from L_β_ to L_α_. We also believe the physical mechanism acting on these two systems to be ultimately the same, i.e., a decrease in lateral pressure. This might be due to structural changes of *Ns*XeR, which under this hypothesis would reduce its volume upon illumination.

Applying the same knowledge to nanodiscs containing *Rs*C*c*O does not lead to the same mechanistic interpretation. The ester band of both DPPC, ^13^C-DPPC and of the PCs in the *E. coli* lipid mixture always shows a positive peak upon oxidation of the enzyme. The appearance of a narrow positive band in the absence of a corresponding negative band can be explained by band narrowing, as an emergent statistical phenomenon in which the activation of *Rs*C*c*O somehow forces a more well-defined thermodynamical state on some or all of the lipids. However, while the peak position around 1,740—1,745 cm^−1^ is indicative of tighter packing of the lipids, this would usually entail the rise of another similarly narrow band centered near 1,715 cm^−1^ (Mantsch et al., 1985; Denisov et al., 2005; Lewis and McElhaney, 2013). It is difficult to comment on the presence of this band based on the data shown in Figure 6, since C*c*Os also show bands around this frequency under certain experimental conditions (Heitbrink et al., 2002; Iwaki et al., 2002; Koutsoupakis et al., 2011). If this band was actually to be found in the positive feature shown between 1,715—1,700 cm^−1^ by *Rs*C*c*O in DPPC and *E. coli* lipids, the interpretation would indeed be clear-cut and involve a transition from the gel phase L_β_ (in which the sample is prepared) to the low-temperature gel state (Mantsch et al., 1985). *Rs*C*c*O would have to only slightly increase its transmembrane volume in order to force a relatively high proportion of DPPC lipids into this tightly-packed state. As mentioned above, we cannot confirm this assignment yet. If no low-frequency band is found, the interpretation will have to involve both a change in packing state and in hydrogen bonding (Lewis et al., 1994) to justify the positive ester band. In fact, the ester band maxima at around 1,740 cm^−1^ in our experiments are most likely representative of a situation in which the solvent environment remains constant (Blume et al., 1988), while a different lipid packing state is transduced by the activation of a membrane protein, which would also be the only source of any H-bonding changes sensed by the lipid heads. The specific protein-lipid interactions in nanodiscs seem to be opposite for *Rs*C*c*O as compared to *Ns*XeR, involving the formation of a tighter lipid packing state upon activation of the former, and a looser packing state in the light-reaction of the latter.

The possibility of any contribution arising from lipids tightly bound to *Ns*XeR (Shevchenko et al., 2017) or *Rs*C*c*O (Svensson-Ek et al., 2002) already in the protein preparation (so-called endogenous lipids) can be excluded from our isotopic substitution experiments. The shift of the whole ester band upon ^13^C/^13^C lipid substitution shows that all lipids which contribute to the signal have been successfully labeled. When it comes to *Ns*XeR, the shifts of the methyl bands support this conclusion. However, it remains to be clarified whether the tension induced by membrane protein activity affects the whole lipid bilayer homogenously, or only a boundary layer in the vicinity of either the scaffold or the integral membrane protein.

Lastly, we showed that nanodisc systems containing reconstituted integral membrane proteins report on secondary or tertiary structure changes upon activation, and they do so by changing their packing state as a consequence of strain being applied to the bilayer. With the help of AzoPC, the lateral tension within the nanodiscs can be remotely elicited and controlled by light. The associated isomerization affects the lipids in the nanodiscs, and finally influences the structure of the scaffold protein. It is tempting to assume that this generic causality applies to the reconstituted membrane proteins as well. In this case though the exact origin of the lateral pressure, possibly related to volume changes, different amphiphilic conditions, or more complex effects including buffer electrostatics, is still unknown and will be a matter for our future studies.

Membrane tension is a major parameter that acts on the activity of membrane proteins. Mechanosensitive channels open and close in response to changes in the lateral pressure of the membrane and are responsible for gravitropism and turgor sensing in plants, contractility of the heart, as well as sensing pain, hearing, and touch in animals (Kefauver et al., 2020). With the techniques presented in this work, the influence of tension can be elucidated by using AzoPC as a light-activatable tool for tension modulation, and nanodiscs not only as biomimetic membranes, but as a mean to generate lateral pressure thanks to the constraint provided by the scaffold protein to the volumetric changes of AzoPC.

To sum up, we demonstrated in this work that catalytically relevant structural changes of membrane proteins influence the lipid membrane in which these proteins are embedded. While this study focused on steady-state FTIR difference spectroscopy, the high temporal and local resolution achievable with IR spectroscopy (Ataka et al., 2010) will permit experiments that target the interaction between protein and membrane on a sub-millisecond time scale. By these means, the dynamic correlation between lipid packing and specific protein states will be addressed.

## Data Availability

The original contributions presented in the study are included in the article/Supplementary Material, further inquiries can be directed to the corresponding authors.

## Abbreviations

ATR, Attenuated total reflection; AzoPC, 1-stearoyl-2-[(E)-4-(4-((4-butylphenyl)diazenyl)phenyl)butanoyl]-sn-glycero-3-phosphocholine; CA, Cardiolipin from *E. coli*; C*c*O, Cytochrome *c* oxidase; DDM, n-Dodecyl-ß-maltoside; DPPC, 1,2-dipalmitoyl-sn-glycero-3-phosphocholine; DMPC, 1,2-dimyristoyl-sn-glycero-3-phosphocholine; FTIR, Fourier Transform Infrared; MSP, Membrane Scaffold Protein; IPTG, Isopropyl β-D-1-thiogalactopyranoside; NaDT, Disodium dithionite; *Ns*XeR, Xenorhodopsin from *Nanosalina* sp; OD600, Optical density at 600 nm; PC, Phosphatidylcholine; PE, L-α-phosphatidylethanolamine from *E. coli*; PG, L-α-phosphatidylglycerol from *E. coli*; POPC, 1-palmitoyl-2-oleoyl-glycero-3-phosphocholine; *Rs*C*c*O, Cytochrome *c* oxidase from *R. sphaeroides*.
