# Supplementary material for: Membrane Protein Activity Induces Specific Molecular Changes in Nanodiscs Monitored by FTIR Difference Spectroscopy
Source: Front Mol Biosci. 2022 Jun 13;9:915328. doi: 10.3389/fmolb.2022.915328 (PMC9234331; doi:10.3389/fmolb.2022.915328)
Supplement: Supplementary file 1 [file DataSheet1.docx]

***Supplementary Material***

**Table S1: Conditions for the reconstitution of integral membrane proteins into nanodiscs.** For the reconstitution, detergent-solubilized membrane proteins were incubated with lipids and membrane scaffold protein (MSP1D1) at defined stoichiometric ratios in 20 mM sodium cholate, 100 mM NaCl, 20 mM TRIS/HCl, under indicated incubation conditions. To initiate the self-assembly of the nanodiscs, SM2 Bio-Beads were added in a w/w ratio of 120% Bio-Beads to the reconstitution mixture to remove the detergent (individual conditions are indicated).

| ***Membrane Protein*** | ***Lipid*** | ***Preparation ratios: Protein/Scaffold/Lipid*** | ***Incubation*** | ***Detergent-removal*** |
| --- | --- | --- | --- | --- |
| - | AzoPC +DPPC / ^13^C_40_-DPPC | 0 / 2 / 150 (DPPC) + 30 (AzoPC) | 1 h at 37°C | Overnight at 4°C |
| *Ns*XeR | DPPC / ^13^C_40_-DPPC | 1 / 2 / 110 | 30 min at 37°C | 30 min at 37°C |
| *Ns*XeR | DMPC | 1 / 2 / 110 | 1 h at 25°C | 2 h at 25°C |
| *Rs*C*c*O | DPPC / ^13^C_40_-DPPC | 1 / 4 / 340 | 30 min at 37°C | 30 min at 37°C  + overnight at 4°C |
| *Rs*C*c*O | *E. coli* polar extract | 1 / 4 / 280 (*E.coli* lipids quantity based on mw estimation) | 30 min at 25°C | 30 min at 25°C  + overnight at 4°C |
| *Nm*HR | DPPC | 1 / 2 / 80 | 30 min at 37°C | 30 min at 37°C |
| *Np*SRII | DMPC | 1 / 2 / 140 | 1 h at 25°C | 2 h at 25°C |
| *Um*RhI | POPC | 1 /2 / 170 | 1 h at 4°C | Overnight at 4°C |


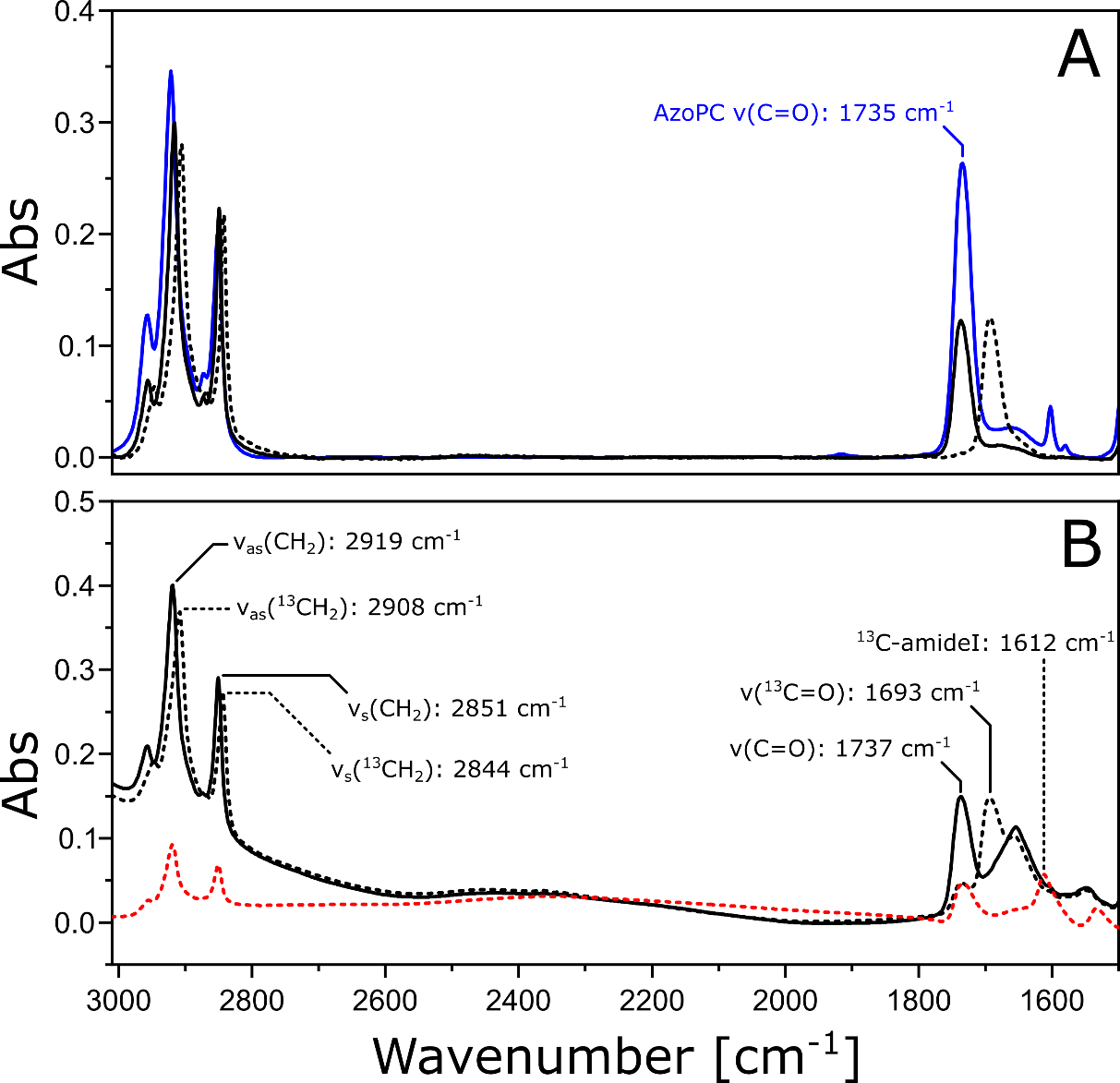


**Figure S1: Absolute spectra of pure lipids and Nd assemblies with AzoPC.** (A) FTIR absorption spectra of *trans*-AzoPC (solid blue line), DPPC (solid black line), and ^13^C-DPPC (dashed black line), dry from CHCl_3_ and deposited on ATR. The spectra were baseline-corrected with an asymmetric least square smoothing baseline. (B) FTIR absorption spectra of the nanodiscs assemblies of 80% DPPC/20% AzoPC with MSP1D1 (solid black line), 80% ^13^C-DPPC/20% AzoPC with MSP1D1 (dashed black line), and 80% DPPC/20% AzoPC with ^13^C-MSP1D1 (dashed red line). The spectra were recorded in transmission mode. Significant vibrational bands are labeled.


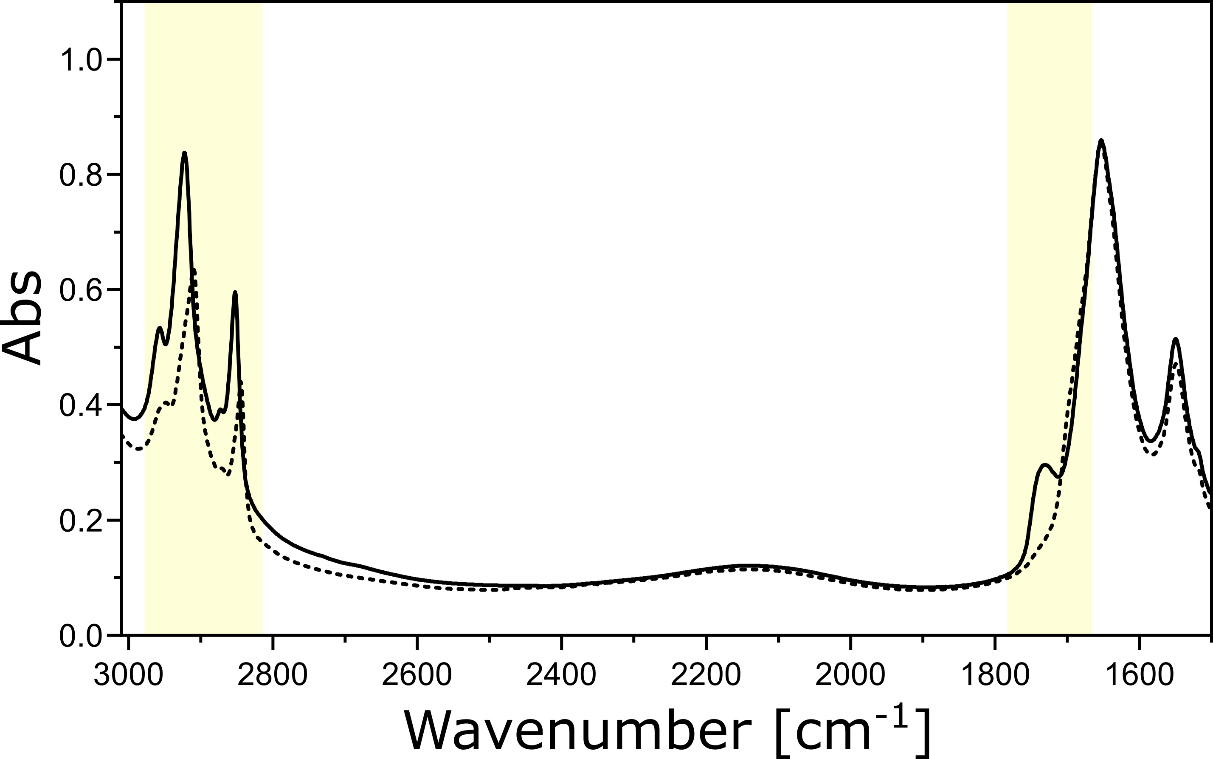


**Figure S2: Absolute spectra of rehydrated xenorhodopsin from the nanohalosarchaeon *Nanosalina*.** Wild-type *Ns*XeR was reconstituted in nanodiscs containing DPPC and ^13^C-DPPC as described in the main text. The spectra of the rehydrated samples in DPPC nanodiscs and in ^13^C-DPPC are shown here as solid and dashed lines, respectively. The frequency regimes of the ester ν(C=O) bands and the alkyl ν(-C-H) of the ^12^C-lipid and its ^13^C-isotopologue are highlighted in yellow. The exposure of these samples to LED illumination resulted in the *Ns*XeR difference spectra reported in the main text.


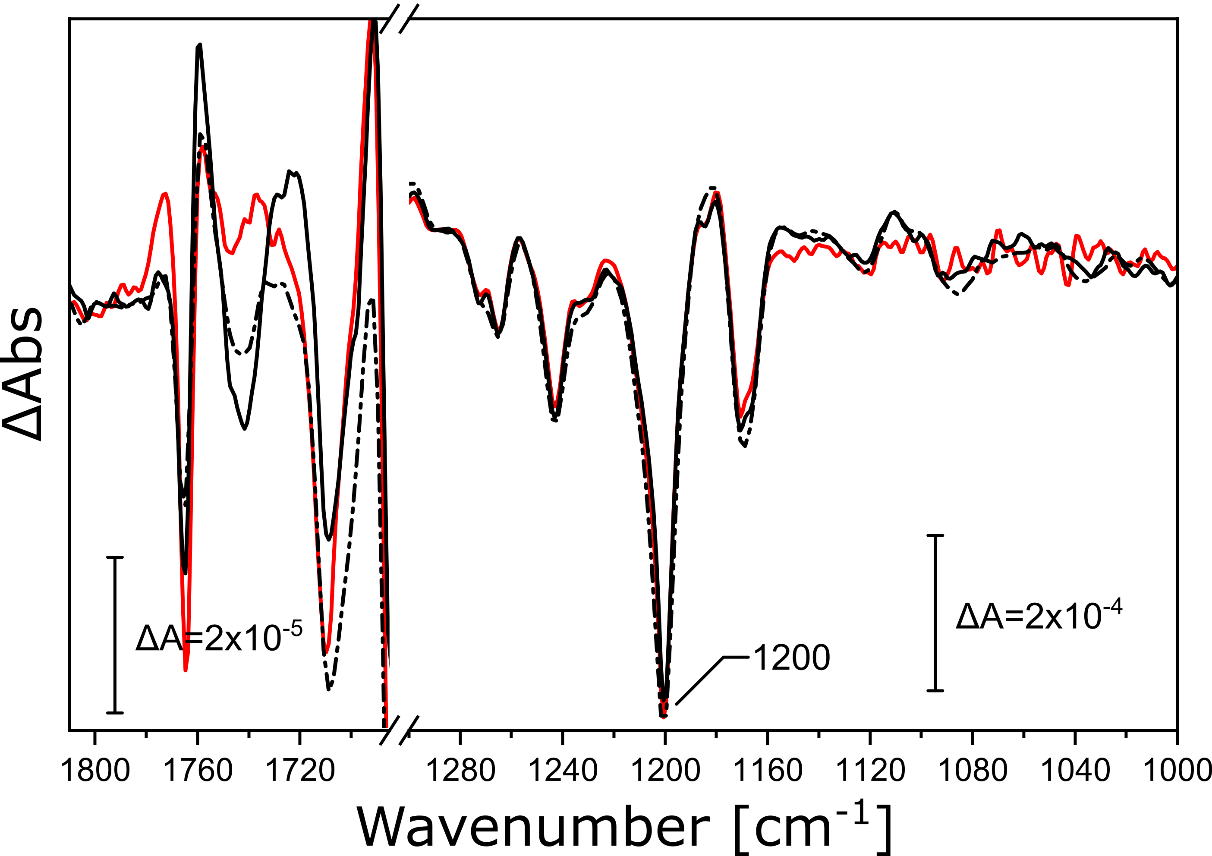


**Figure S3: Comparison between the effects of different lipids and detergent on *Ns*XeR.** Light *minus* dark difference spectra upon 525 nm LED illumination of *Ns*XeR in nanodiscs containing DPPC (solid black line, same dataset as **Figure 4** in the main text), DMPC (dash-dotted black line), or *Ns*XeR solubilized with DDM (solid red line, same dataset as the inset in **Figure 4** of the main text). The spectra are normalized to the 1200 cm-1 peak of the retinal ν(C-C).


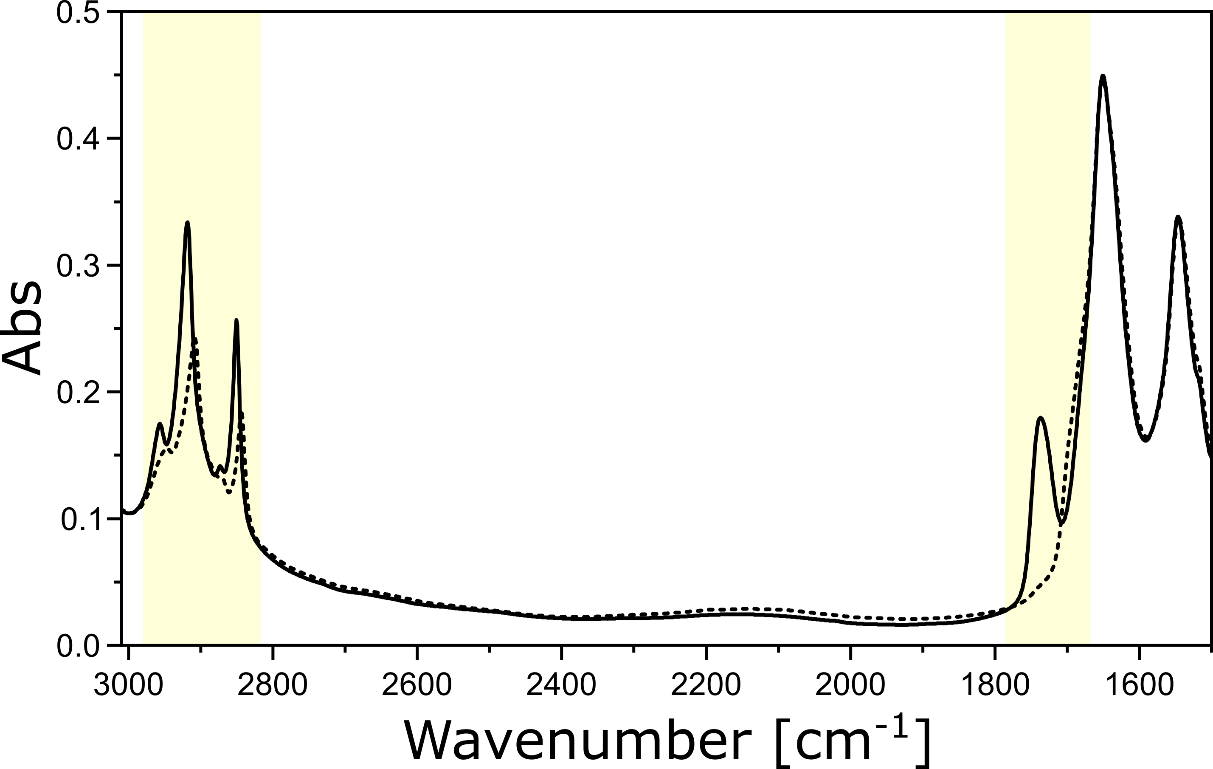


**Figure S4: Absolute spectra of rehydrated Cytochrome *c* Oxidase from *R. sphaeroides*.** *Rs*C*c*O was reconstituted in nanodiscs containing DPPC and ^13^C-DPPC as described in the main text. The samples were dried on ATR, reduced by NaDT, and subsequently rehydrated by H_2_O aerosol in N_2_ carrier. The spectra of the rehydrated samples in DPPC nanodiscs and in ^13^C-DPPC are shown as solid and dashed lines, respectively. The frequency regimes of the ester ν(C=O) bands and the alkyl ν(-C-H) of the ^12^C-lipid and its ^13^C-isotopologue are highlighted in yellow. Reacting these samples with O_2_ resulted in the difference spectra presented in **Figure 6** of the main text.


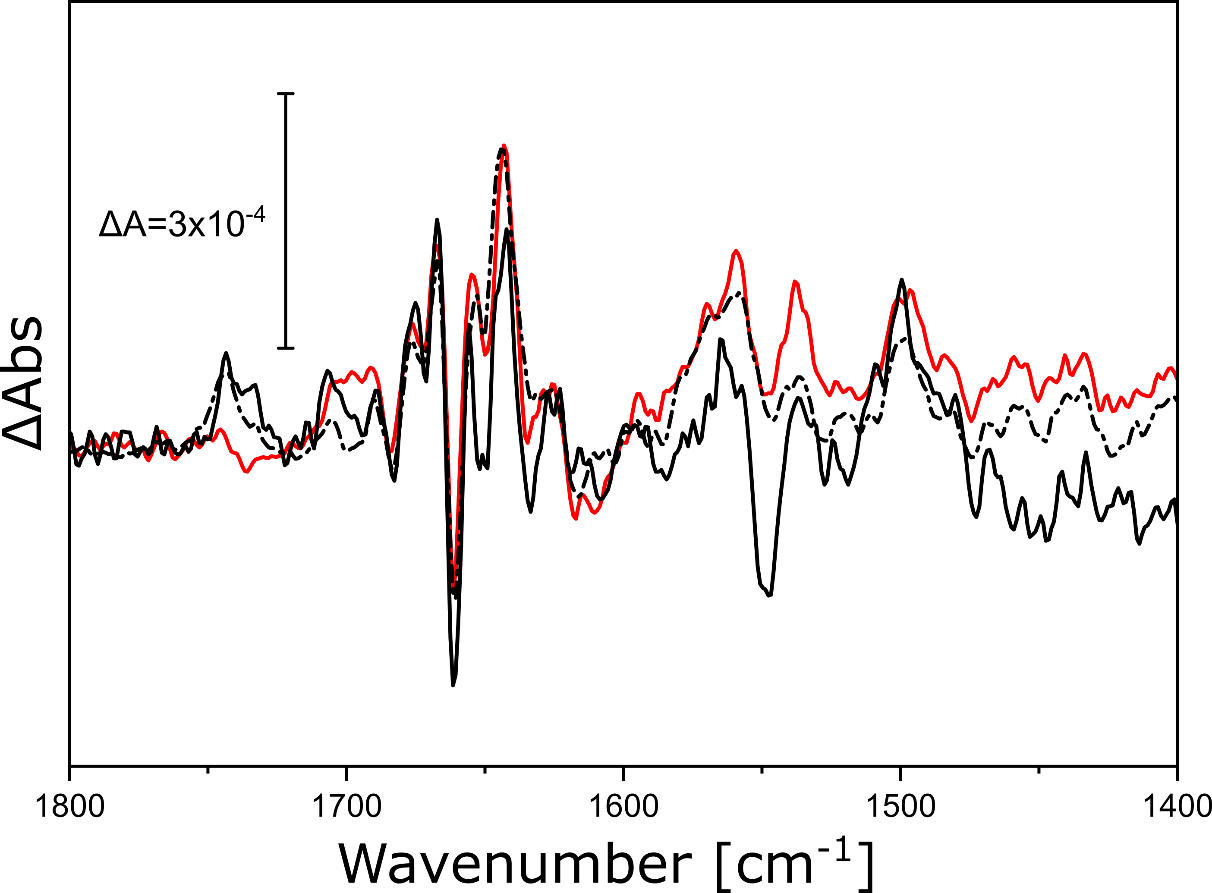


**Figure S5: FTIR difference spectra of *Rs*C*c*O reconstituted in nanodiscs with different lipids.** The same datasets as in **Figure 6** of the main text are shown in a broader frequency regime in order to facilitate comparison between the response of different samples. O_2_-oxidized *minus* NaDT-reduced difference spectra of *Rs*C*c*O in nanodiscs with DPPC, ^13^C-DPPC and *E. coli* polar lipids are shown, respectively, as solid black, solid red, and dash-dotted black lines.


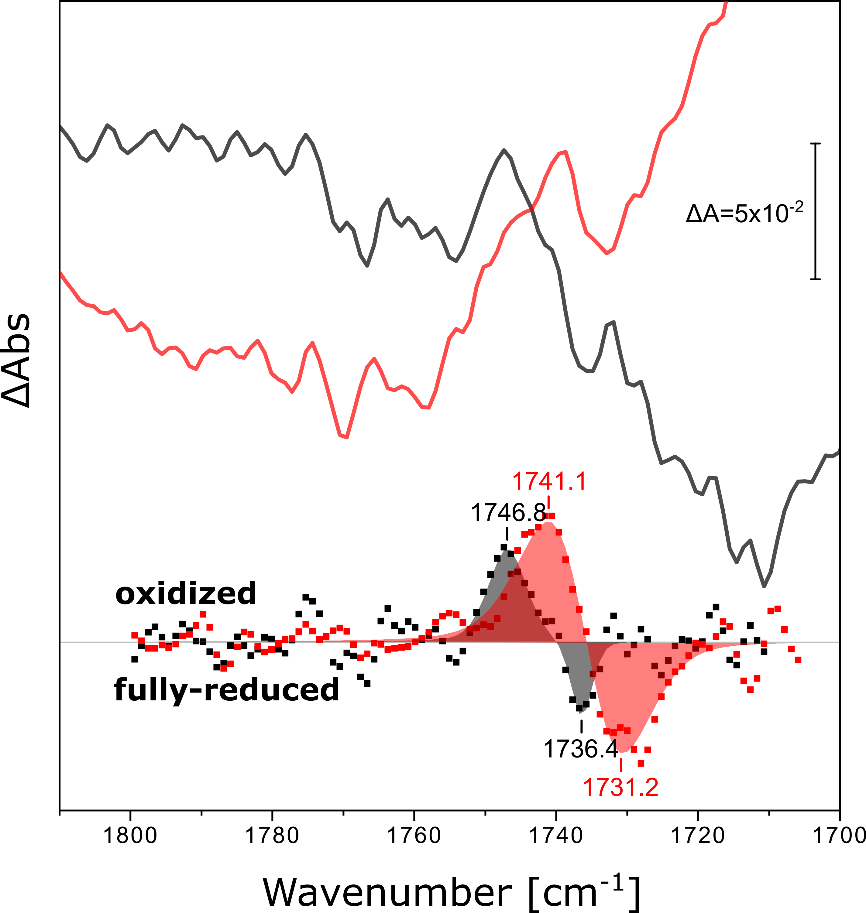


**Figure S6: Oxidized minus fully-reduced FTIR difference spectra of *Rs*C*c*O in DDM.** The “raw” difference spectra of DDM-solubilized *Rs*C*c*O in H_2_O and D_2_O were recorded in the same way as described in the main text and are shown as solid grey and solid red lines, respectively. The bottom dotted traces represent the same spectra upon baseline correction with a polynomial spline (H_2_O data as black squares, D_2_O data as red squares). The baseline-corrected spectra were fitted with 2 Voigtians for each spectrum (shaded areas). Peak positions belonging to the bands of E286 are indicated.


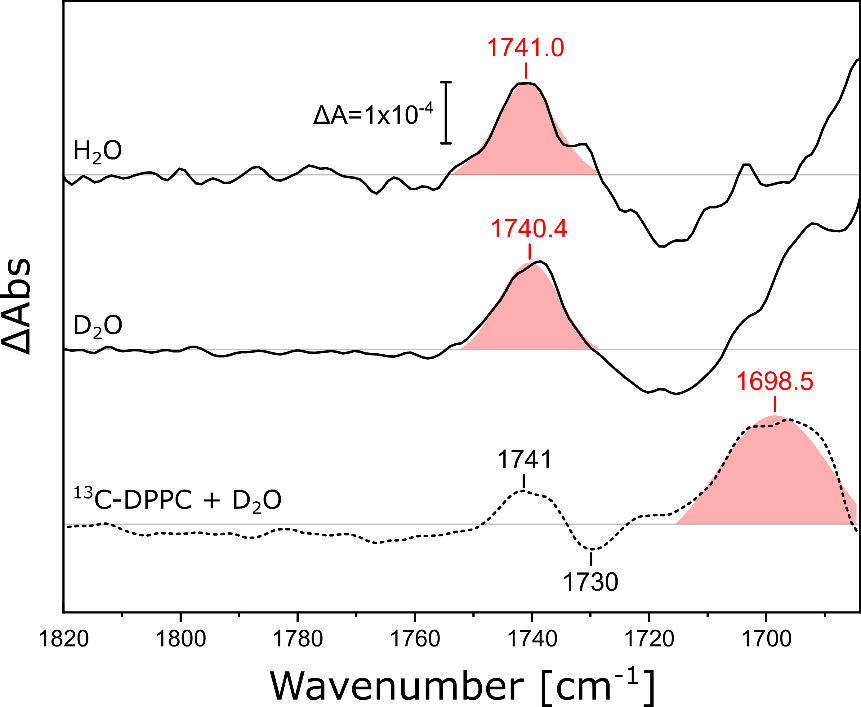


**Figure S7: Potential-induced difference spectra of *Rs*C*c*O.** From top to bottom: potential-induced difference spectra of oxidized minus fully-reduced *Rs*C*c*O in DPPC and H_2_O (solid black line), DPPC and D_2_O (solid black line), and ^13^C-DPPC and D_2_O (dashed black line). The spectra were recorded in lipid “cakes” on ATR as described previously (Baserga et al., 2021). Voigtian fits are shown as red shaded areas. The top and bottom spectra are mirrored and replotted from Figure S7 of (Baserga et al., 2021). Fitted peak positions are highlighted by red labels. Peak positions corresponding to the band shift of E286 are indicated in black. Zero lines are shown in grey.

**References**

Baserga, F., Dragelj, J., Kozuch, J., Mohrmann, H., Knapp, E.-W., Stripp, S.T., et al. (2021). Quantification of Local Electric Field Changes at the Active Site of Cytochrome *c* Oxidase by Fourier Transform Infrared Spectroelectrochemical Titrations. *Front. Chem.* 9. doi: 10.3389/fchem.2021.669452.
